# Supplementary material for: Overcoming stability challenges during continuous intravenous administration of high-dose amoxicillin using portable elastomeric pumps
Source: PLoS One. 2019 Aug 16;14(8):e0221391. doi: 10.1371/journal.pone.0221391 (PMC6697341; doi:10.1371/journal.pone.0221391)
Supplement: Attachment — Submitted filename: Binson et al 2019 Response to Reviewers.docx [file pone.0221391.s001.docx]

**Pr Antoine DUPUIS**

Pharmacien, PU-PH

Faculté de Médecine et de Pharmacie

6, rue de la Milétrie

86034 Poitiers

antoine.dupuis@univ-poitiers.fr

Axe HEDEX

Inserm CIC 1402

Pharmacie

CHU de Poitiers

2 rue de la Milétrie

CS 90577

86021 Poitiers cedex

a.dupuis@chu-poitiers.fr

🕿 : 05 49 44 37 68

**To: Editor-in-chief**

**PLOS ONE**

Poitiers, 6^th^ august 2019

Dear Doctor,

Please find enclosed our revised manuscript (PONE-S-19-21718), entitled "Overcoming stability challenges during continuous intravenous administration of high-dose amoxicillin using portable elastomeric pumps" by Guillaume Binson, Claire Grignon, Gwenaël Le Moal, Pauline Lazaro, Jérémy Lelong, France Roblot, Nicolas Venisse and Antoine Dupuis.

We sincerely appreciate the comments of the reviewers and revised the paper according to the suggestions. Please find below the details point-by-point of the revisions in the manuscript and our responses.

Yours faithfully,

Pr Antoine Dupuis

Pharm.D., Ph.D.

Université de Poitiers, Faculté de Médecine et de Pharmacie, CIC Inserm 1402

Tel: +33 5 49 44 37 68; fax:+33 5 49 44.39.72

E-mail: antoine.dupuis@univ-poitiers.fr

**Corresponding author**

Pr Antoine Dupuis,

School of Pharmacy, University of Poitiers, CIC Inserm 1402

6, rue de la Milétrie, 86034 Poitiers, France

Tel: +33 5 49 45 43 26; fax:+33 5 49 45.43 05

E-mail address: [antoine.dupuis@univ-poitiers.fr](mailto:antoine.dupuis@univ-poitiers.fr)

**Response to reviewers**

**Reviewer #1**

“Excellent manuscript. The methodology follows the recommendations for drug stability testing of the British Society of Antimicrobial Chemotherapy. The discussion includes a complete review of the existing literature and addresses all the important issues around the problem of drug stability in elastomeric pumps.”

We sincerely appreciate the comments of the reviewer.

**Reviewer #2**

“This paper describes the results from an experimental research work that aim to study the stability of amoxicillin intravenous preparations in elastomeric pumps. The study was well-designed and the results are presented clearly and are adequately discussed. This work is of interest for clinical practice. Some minor improvements can be considered:»

Thank you very much for your valuable and constructive suggestions. We have carefully revised the manuscript according to the suggestions

“1 - in the methods section, regarding the conditions for stability testing on Climate Chambers (line 110) it should be specified if the humidity was also controlled.”

According to reviewer’s suggestion, the sentence has been modified as follow: “The filled elastomeric pumps were stored at 25 ± 1°C for 24 hours in a climate chamber **without humidity control** (Air concept®, FirLabo, France).”

“2 - in the conclusion section (line 316) the information "elastomeric pump may be stored at ambient temperature for 12 hours" should be completed with the specific range of temperature related to the conclusion.”

According to reviewer’s suggestion, the range of temperature has been added: “Then, under these optimized conditions, the elastomeric pump may be stored at ambient temperature **(22 ± 4°C)** for 12 hours…”

“3 - the last sentence of the conclusion (lines 320-322) is not clear: it seems to suggest that the elastomeric pump is kept refrigerated during administration. Although these pumps may be refrigerated for storage, drug administration is generally preceded by warming the system at room temperature. Also, according to the presented stability data if the elastomeric pump was kept refrigerated for 24h it would not be expected to be stable for an additional period of 12h at room temperature (corresponding to a loss of drug of approximately 17% of drug). This should be clearly discussed.”

The sentence has been modified in order to clarify this point : “Finally, amoxicillin may remain stable over the entire day under the same conditions, provided that the elastomeric pump is stored between +4°C and +8°C **throughout the administration**, using for instance a refrigerated bag ; **however this can lead to potential intolerance due to infusion of cold solution**.
